# Prediction of Mechanical Properties of Lattice Structures: An Application of Artificial Neural Networks Algorithms

**DOI:** 10.3390/ma17174222

**Published:** 2024-08-27

**Authors:** Jiaxuan Bai, Menglong Li, Jianghua Shen

**Affiliations:** 1 School of Transportation Science and Engineering, Civil Aviation University of China, Tianjin 300300, China; 15398097198@163.com; 2 School of Aeronautics, Northwestern Polytechnical University, Xi’an 710072, China; menglongli@mail.nwpu.edu.cn

**Keywords:** lattice structure, artificial neural networks, Young’s modulus, yield strength

## Abstract

The yield strength and Young’s modulus of lattice structures are essential mechanical parameters that influence the utilization of materials in the aerospace and medical fields. Currently, accurately determining the Young’s modulus and yield strength of lattice structures often requires conduction of a large number of experiments for prediction and validation purposes. To save time and effort to accurately predict the material yield strength and Young’s modulus, based on the existing experimental data, finite element analysis is employed to expand the dataset. An artificial neural network algorithm is then used to establish a relationship model between the topology of the lattice structure and Young’s modulus (the yield strength), which is analyzed and verified. The Gibson–Ashby model analysis indicates that different lattice structures can be classified into two main deformation forms. To obtain an artificial neural network model that can accurately predict different lattice structures and be deployed in the prediction of BCC-FCC lattice structures, the artificial network model is further optimized and validated. Concurrently, the topology of disparate lattice structures gives rise to a certain discrete form of their dominant deformation, which consequently affects the neural network prediction. In conclusion, the prediction of Young’s modulus and yield strength of lattice structures using artificial neural networks is a feasible approach that can contribute to the development of lattice structures in the aerospace and medical fields.

## 1. Introduction

Lattice structures are a type of structure constituted by periodic or quasi-periodic lattice units, which possess a distinctive geometry that enables them to be lightweight, exhibit high specific strength, demonstrate high energy absorption, and exhibit excellent thermal conductivity. Within the modern engineering field, the applications of lattice structures are very wide, including a variety of industries such as aerospace, automotive manufacturing, and biomedical [1,2,3]. In the aerospace sector, lattice structures achieve significant weight reductions to improve the fuel efficiency and load capacity of aircraft by optimizing the distribution of materials [4,5,6]. The elastic modulus and strength of the lattice structure units are lower than those of the general matrix material, and titanium alloy has good biocompatibility. Additively manufactured titanium alloy composite lattices have been used as human implants to match the human body by elastic modulus and strength to avoid stress shielding effect [7]. Simultaneously, its porosity helps to improve the growth of bone tissue from the inside to the outside, and the open lattice structure is more conducive to the use of antibiotics to control and eliminate possible infection [8]. The automotive industry is also benefiting from the applications of lattice structures, especially in the design of electric vehicles. Lattice structures can not only be used in the lightweight design of the body but also used for the battery pack cooling system to improve heat-pipe efficiency and extend battery life to enhance vehicle performance. Lattice structures, with their distinctive characteristics, have the potential to be applied in several industrial fields, thereby contributing to the advancement of materials science and engineering technology.

Given the extensive range of applications for lattice structures, the design of lattice structures that can be used in different situations has become a significant area of research for those working in this field. The prevailing design methodologies for lattice structures encompass topology optimization, parametric design, and experiment-based design techniques. These methodologies entail the manipulation of the geometric parameters of the lattice structure, material distribution, and other variables to achieve lightweight, high strength, and other performance. M. R. Costa et al. [9] proposed a two-scale concurrent optimization of the lattice structure, where the topology optimization at the macro-scale structure and the underlying material micro-structures is performed simultaneously to achieve optimal topologies. Long Chen et al. [10] proposed a volume parametric modeling method of lattice structures and realized an efficient multiscale topology optimization framework based on iso-geometric analysis (IGA) to construct the gradient lattice structure. The topology optimization analysis requires a significant amount of computation, particularly when handling complex geometric forms [11,12,13,14]. Moreover, topology optimization has the checkerboard pattern effect, and generated structural states are complex, which is difficult to manufacture. Parametric design allows the user to define a group of geometric parameters, including unit size, geometry, permutation, and so forth. These parameters can then be varied to produce different lattice structures [15,16,17]. This method is flexible and easy to modify, but the range of selectable parameters is considerable. Consequently, the researcher must undertake a significant number of experiments and iterations to identify the optimal structure, which is the cause of the low efficiency.

In recent times, the field of machine learning has witnessed a remarkable surge in advancement, which has brought new opportunities for the design of the lattice structures. In contrast to the traditional approach, machine learning enables the efficient prediction and optimization of lattice structures through the handling of a substantial quantity of design data and the learning of complex design patterns. Currently, there is a body of research investigating the use of related neural networks for the prediction of structural designs. For example, Chang Liu et al. proposed a novel 3D convolutional neural network (3D-CNN) with transfer learning (TL) to predict the performance of lattice structures [18]; Denis Horvath et al. achieved the intrados self-energy prediction using the neural network [19]; Y. Pan et al. investigated the identification problem for spatiotemporal patterns that are generated by autonomous cellular neural networks (CNN) [20]. With the development of deep learning, graph neural networks (GNN) was proposed. Researchers have used GNN models to make successful predictions on various aspects, such as crystal materials and mechanical structures [21,22,23]. Up to now, the existing graph construction methods are insufficient, and the data may have poor quality with outliers, uncertain connections, and missing values, considering that the quality of the graph has a large impact on the performance of the GNN model [24]. There is no denying that the utilization of machine learning approaches for the prediction of lattice structures holds significant feasibility, offering high design efficiency and the ability to explore a broader design space, thereby advancing the application and development of lattice structures across various domains.

Through the above discussion, the main work of this paper is summarized as follows:

(1) For the prediction of structural mechanical properties, researchers usually use the equivalent voxel method [18] to construct the dataset, which is usually characterized by a large amount of input data and unclear relationships between the data. Therefore, this paper will be based on the topology between different lattice structures, which can greatly reduce the size of the input dataset and ensure validity at the same time.
(2) Meanwhile, this paper is based on experimental results and adopts the finite element calculation method, which greatly expands the sample size of the dataset and helps the accuracy of the validation. Considering the maturity of current neural network research, this paper adopts the artificial neural network method to ensure the reliability of the results.
(3) This prediction method classifies the structure of Gibson–Ashby model for the first time. And based on the classified models, machine learning is carried out separately, and better prediction results are finally obtained. It can provide directional support for subsequent lattice structure optimization design.

Overall, in this study, different structures of BCC-FCC were established by finite element calculations. The neural network was then used to predict the yield strength and Young’s modulus (the open-source code will be released at https://github.com/leemenglong/ANN-for-Lattice-Structure.git (accessed on 21 August 2024)), thereby proposing new research directions for lattice structures in the medical and structural mechanical sectors. 

## 2. Method

### 2.1. Artificial Neural Network Algorithm

An artificial neural network (ANN) is a computational model that emulates the structural and functional characteristics of biological neural networks. It is particularly adept at processing complex, non-linear data [25]. By connecting layers of neurons, the ANN is able to learn and extract features from large amounts of data and discover hidden patterns and relationships. In the design of lattice structures, the ANN is able to predict the performance of the new structures through considerable training on known structures and data on their mechanical properties, thereby improving design efficiency and precision. Consequently, its capacity for handling data and prediction enables it to be an effective instrument for resolving intricate issues.

**Figure 1:**
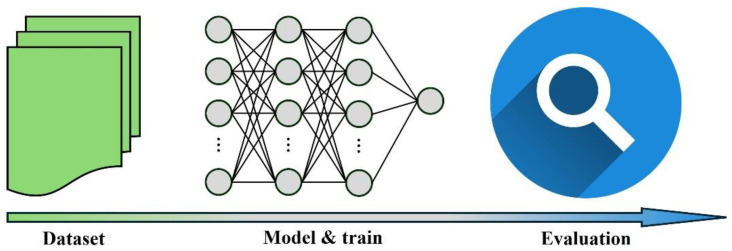
The predictive workflow of the artificial neural network.

The construction of neural networks is typically uniform and usually needs to be built in the following steps [26,27]: (1) dataset construction; (2) model selection and architecture design; (3) model initialization and training; and (4) model validation and optimization. The predictive workflow of the artificial neural network is shown in Figure 1.

### 2.2. ANN Dataset

In the current research for machine learning and lattice structure design optimization, there are two main ways to set up the dataset for machine learning:

(1) Geometric features of a single lattice structure are selected as input. For example, Xianglong Peng’s research [28] goal is triangular lattice structures, which he treats as equivalent homogeneous materials, and uses the strut thickness t, the unit cell width ω, and the strut angle θ as feature inputs, which are only useful for his research on triangular lattice structures and cannot be generalized to other models. Guoji Yu [29] composites rhombic dodecahedron (RD) and octet-truss (OT) unit cells, treating RD and OT as separate feature inputs, a method that can also only be used for the unique material structures of this literature. Chundi Zhang [30] similarly uses the angle and thickness of the material as input, which has some limitations.

(2) Digital visualization of geometric features. For example, Chi Wu [31] maps the 3D lattice structure directly into an 80 × 80 × 80 brick element for input computation, and Asuka Suzuki [32] divides the lattice structure into 20 cross-sections, and each cross-section draws 20 feature lines for input computation. This mapping method will have a larger amount of input features.

Unlike the above work, in the work of this paper, the structural topology will be used as the parameter input, which can be computed and predicted for a wide range of lattice structure models and is more generalizable. Moreover, the method has fewer parameter inputs, stronger relative independence between parameters, less computational resource consumption, and faster fitting speed.

**Figure 2:**
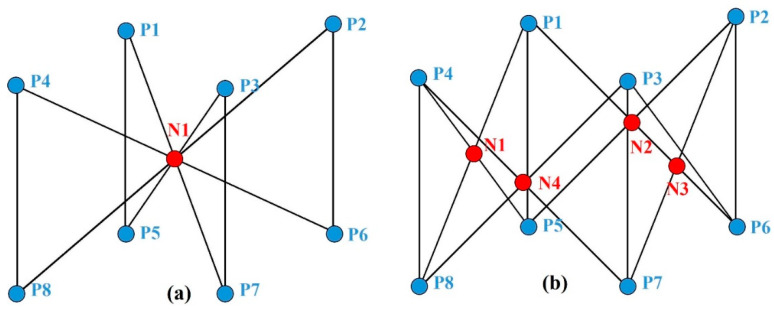
(**a**) BCCZ and (**b**) FCCZ two typical lattice structures.

The Young’s modulus and yield strength of the lattice structures are closely related to the topology of that lattice unit. Consequently, in this study, the coordinates of node units in the form of a lattice structure and point-to-point connectivity are employed as the input dataset for machine learning. In the literature of Miao Zhao [3], the mechanics were revealed through the research of 32 different lattice structures of BCC. A typical BCC structure with a cell length of 4 mm, a cross-section diameter of 0.412 mm, and a relative density of 0.16 with materials Ti-6Al-4V is defined in the article. The calculations in this study will be based on the same material and the same cell length and cross-sectional area. The definition of datasets is based on the lattice structures depicted in Figure 2.

The lattice pillars of the lattice structures have a diameter of 0.412 mm and a single cell edge length L of 4 mm. For each different lattice structure, the nodes of its single cell are divided into two groups: fixed-existing nodes (blue nodes in Figure 2, P1–P8) and movable nodes (red nodes in Figure 2, N1–N4). As the positions of nodes P1-P8 are fixed, they are not included in the training dataset. Consequently, the node coordinate dataset for variable N1-N4 nodes is defined as $M_{L}$:

(1) $$\begin{array}{c} M_{L}=\frac{\left[X_{N1},Y_{N1},Z_{N1},X_{N2},\ldots ,Z_{N4}\right]}{L} \end{array}$$

A total of 12 coordinate data points is present in the node coordinate dataset, with a data range of [0, 1] as indicated by Equation (1).

Concurrently, it is necessary to define the connections between the points to represent each of the different lattice structures. Define the cell matrix $M_{E}$ with shape 12×12:

(2) $$\begin{array}{c} M_{E}=\left[\begin{array}{ccc} \begin{array}{ccc} E1,1 & E1,2 & E1,3\\ E2,1 & E2,2 & E2,3\\ E3,1 & E3,2 & E3,3 \end{array} & \cdots & \begin{array}{c} E1,12\\ E2,12\\ E3,12 \end{array}\\ \vdots & \ddots & \vdots \\ \begin{array}{ccc} E12,1 & E12,2 & E12,3 \end{array} & \cdots & E12,12 \end{array}\right] \end{array}$$

The $E_{i,j}$ in Equation (2) can be calculated as:

(3) $$\begin{array}{c} Ei,j\;\left\{\begin{array}{c} i,j=1\sim 8\;in\;P1-P8\\ i,j=9\sim 12\;in\;N1-N4 \end{array}\right. \end{array}$$

In Equation (3), if there is a connecting pillar between nodes P1 and P2, then E1,2=1 and vice versa E1,2=0. Similarly, if there is a connecting pillar between nodes P1 and N1, then E1,9=1 and vice versa E1,9=0. The following properties can be derived from the matrix $M_{E}$:

(4) $$\begin{array}{c} \left\{\begin{array}{c} Ei,i=0\\ Ei,j=Ej,i \end{array}\right.\;\left(i,j=1\sim 12\right) \end{array}$$

Consequently, the matrix $M_{E}$ comprises a total of 66 independent parameters. By implementing the following alterations to this matrix, the number of parameters is reduced while maintaining independence.

(5) $$\begin{array}{c} M_{E}^{\prime }=\left[R1,R2,\ldots ,R11\right] \end{array}$$

(6) $$Ri=\frac{\sum_{j=i+1}^{12}Ei,j\times 2^{12-j}}{1+\sum_{n=0}^{12-i}2^{n}}$$

The processed dataset $M_{E}^{\prime }$, calculated by Equations (5) and (6), comprises a total of 11 independent data points, with a data range of [0, 1]. In accordance with Equations (5) and (1), the input dataset of the neural network is defined as DataSet:

(7) $$\begin{array}{c} DataSet=\left[M_{L},M_{E}^{\prime }\right] \end{array}$$

The DataSet model comprises 23 input parameters. Based on the comparison of the research methodology of this work with that of other researchers, Table 1 was obtained.

From the above comparison, this work utilizes the topology of lattice structure for mathematical definition and reduces the feature values to 23, which can greatly reduce the consumption of computational resources to speed up the training speed, and effectively prevent the model from overfitting. Its input features are relatively independent, which can effectively avoid feature redundancy and improve model stability. Moreover, the direct input of topology structure is beneficial for the model to be generalized and applied among different lattice structures; for example, it is applied to Xianglong Peng’s triangular lattice structure [28].

In this study, based on the above form of dataset composition, 1000 subclasses were randomly generated for each of the four different structures with 1 to 4 nodes within the lattice structural unit, respectively, totaling 4000 subclasses for subsequent learning. The form of datasets in Table 2.

### 2.3. Finite Element Method Calculation

The mechanical properties of lattice structures are largely dependent on the matrix material of the lattice. The mechanical property parameters of the matrix materials used in this study are presented in Table 3 [3].

**Figure 3:**
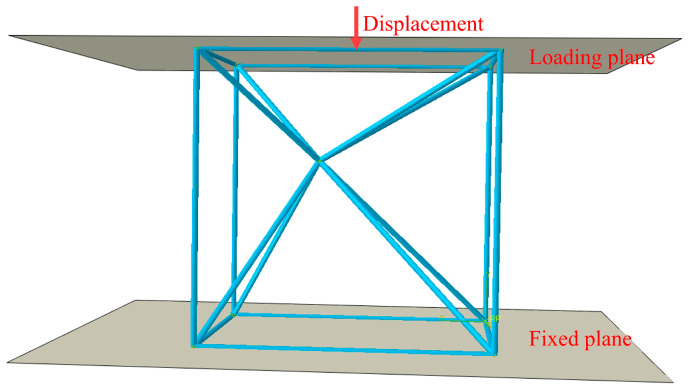
FEM model for simulation.

In this study, ABAQUS was developed in a secondary capacity by Python, with the objective of completing finite element analysis and generating the necessary data for machine learning. Build the finite element model in Figure 3. The mesh size of the point structure is 0.1 mm with Beam cells (B31). The upper and lower parts of the lattice structure have a loading plane and a fixing plane, and a reference point is set in the middle of the upper loading plane for loading displacements and reading reaction forces. During the loading process, the fixed plane was maintained in a fixed position, while the downward compressive displacement of the loading plane was 0.5 mm with a nominal strain of 0.125. This was analyzed quasi-statically using ABAQUS/standard.

## 3. Result and Discussion

### 3.1. Mechanical Properties

**Figure 4:**
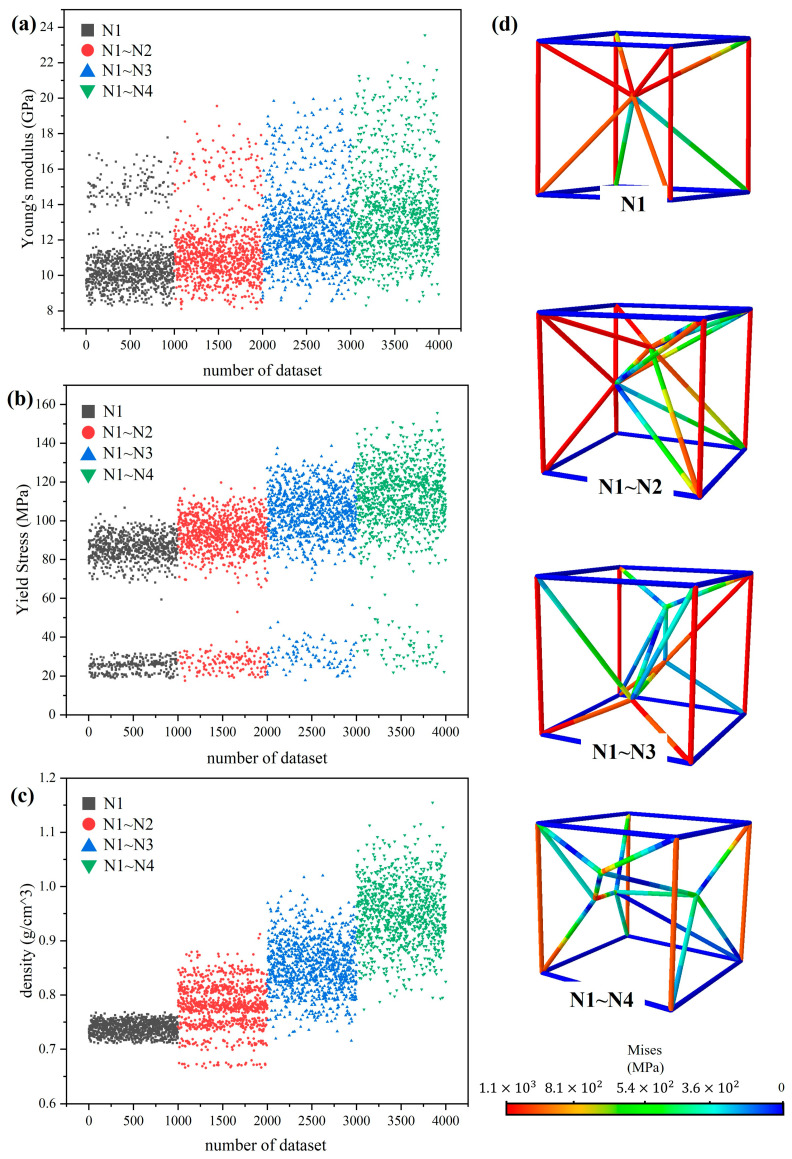
(**a**) Young’s modulus; (**b**) yield strength; (**c**) density of ANN dataset; (**d**) the Mises stress of 4 types of lattice structures.

A finite element analysis was conducted on the 4000 structures generated in the article [3] in order to ascertain the Young’s modulus, yield strength, and density of each structure, as illustrated in Figure 4. The number of internal nodes has a significant effect on the mechanical properties and density of the lattice structure. As the number of internal nodes increases, the Young’s modulus, yield strength, and density of the structure increase to varying degrees. Concurrently, an expansion in the number of nodes engenders an enlargement in the domain of potential lattice structures, thereby engendering a gradual dispersion in the values derived from their calculation.

**Figure 5:**
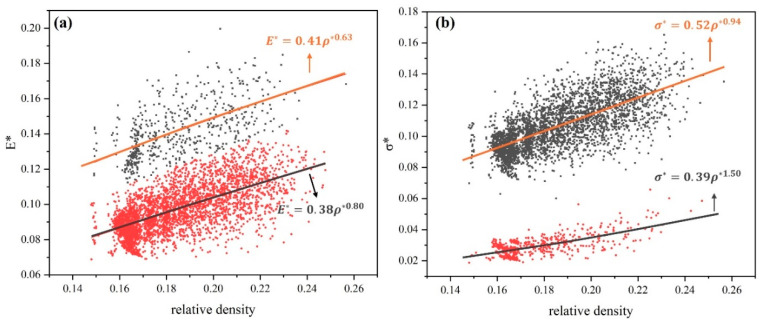
(**a**) Relative Young’s modulus $E^{*}$. (**b**) Relative yield strength $\sigma^{*}$ and relative density $\rho^{*}$. The inserted equations indicated the fitting result.

Figure 4a,b demonstrates a notable grouping of Young’s modulus and yield strength. To elucidate the deformation behavior of these lattice structures, the classical Gibson–Ashby model [35,36,37,38], which correlates relative volume fractions and mechanical properties, was employed. The Gibson–Ashby model is represented by the following equations:

(8) $$E^{*}=\frac{E_{L}}{E_{S}}=A_{1}{\left(\rho^{*}\right)}^{n_{1}}$$

(9) $$\sigma^{*}=\frac{\sigma_{L}}{\sigma_{S}}=A_{2}{\left(\rho^{*}\right)}^{n_{2}}$$

(10) $$\rho^{*}=\frac{\rho_{L}}{\rho_{S}}$$

where $E_{L},\;\sigma_{L},\rho_{L}$ are Young’s modulus, yield strength, and density of the lattice structures; $E_{S},\;\sigma_{S},\;\rho_{S}$ are Young’s modulus, yield strength and density of the matrix material, and $\rho^{*}$ is the relative density. The Gibson–Ashby coefficients $A_{1},\;A_{2}$ with indices $n_{1},n_{2}$ were obtained by fitting the simulated data points. The results are shown in Figure 5. For all samples, the $A_{1}$ and $A_{2}$ coefficients fall within the range of 0.1–4.0 as outlined in Gibson–Ashby. However, it should be noted that the Gibson–Ashby coefficients are not only dependent on the configuration of the lattice structural unit and the type of material used, but also on the direction of the load applied to the lattice structure. 

In Figure 5, the value of $n_{1}$ is the value of 0.80 and the value of $n_{2}$ is the value of 0.94, which implies that this type of lattice structure is mainly dominated by stretching. However, these values are slightly lower than the value of 1 given by Gibson–Ashby for the ideal stretch-dominated structure. Furthermore, in Figure 4, the value of $n_{1}$ is 0.63, while the value of $n_{2}$ is 1.50. In contrast, the value of $n_{1}$ is 1, while the value of $n_{2}$ is 1.5 for the curved dominant lattice structure presented in Gibson–Ashby. In the fitting of Young’s modulus, $n_{1}$ is found to be smaller than the theoretical value, which is attributed to the presence of connected pillars on the Z-axis in this type of lattice structure. This discrepancy from the theoretical value is typically attributed to the lattice design, and such phenomena have been found in articles in Maskery [39] and Haozhang [40].

### 3.2. The Prediction of ANN

The configurations of the different artificial neural networks have some differences in the usefulness of different problems [30]. In the process of learning modeling of yield strength and Young’s modulus, the prediction effect of neural networks with different architectures was observed. Following an appropriate testing and optimization phase, two forms of artificial neural networks were employed for the learning and prediction of structures exhibiting distinct deformations, as illustrated in Figure 5a,b.

The selection of artificial neural network parameters of yield strength is presented in Table 4. During the model training process, the data partitioning strategy of 4000 with an 8:2 ratio of training data to test data ensures the reliability and generalizability of the model prediction results. 

**Figure 6:**
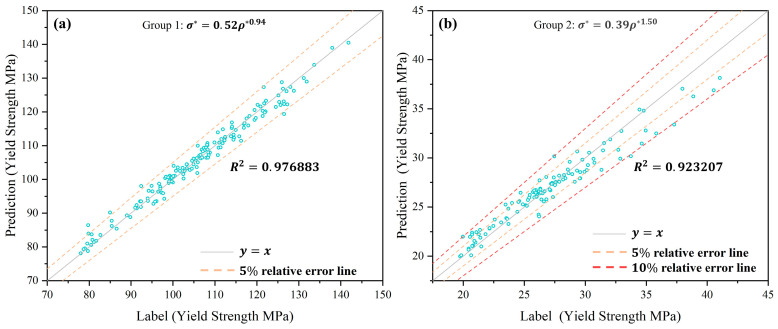
Neural network prediction of yield strength results. (**a**) Group 1; (**b**) group 2.

Following the training of the neural network, the predicted yield strength and the actual yield strength are presented in Figure 6. The comparison reveals that most predicted and actual yield strengths exhibit greater consistency, with only a small number of data points deviating from the predicted values. The distribution of data points exhibiting large deviations is uniform, and there is no discernible phenomenon of “enrichment” in the error distribution. Figure 6a illustrates that most data points lie within the 5% relative error line. Figure 6b illustrates that most of the data points fall within the 5% relative error line, with a smaller number falling within the 10% relative error line. The preceding results demonstrate that the neural network exhibits high accuracy in analyzing and predicting the yield strength of different point structures. 

Furthermore, the coefficient of determination $R^{2}$ is a statistical indicator of the degree of correlation between variables. $R^{2}$ is typically situated within the range [0,1], with a closer proximity to 1 indicating a stronger correlation. Consequently, R is employed to quantify the extent of correlation between the training outcomes and the target values. As illustrated in Figure 6, the correlation coefficients $R^{2}$ between the predicted and trained values for the two datasets are 0.976883 and 0.923207, respectively.

Similarly, the parameters learned by the neural network for Young’s modulus are presented in Table 5, with an 8:2 ratio of training and test data.

**Figure 7:**
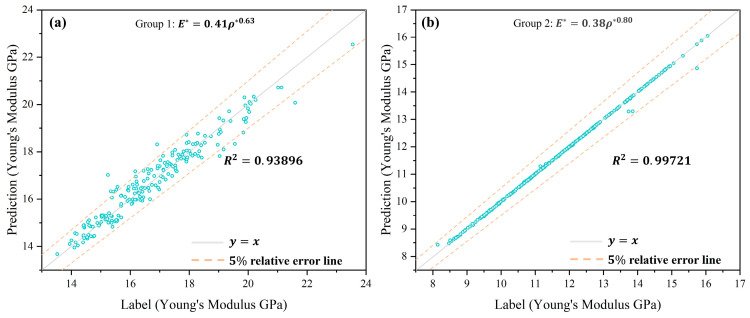
Neural network prediction of Young’s modulus results. (**a**) Group1; (**b**) group 2.

A comparison of the predicted and theoretical results following neural network training is shown in Figure 7. The majority of the predicted and theoretical values are situated along the 45° diagonal line. For the initial set of predicted values, the data points are predominantly located within the 5% relative error line, with a correlation coefficient $R^{2}$ of 0.93896. For the second set, $R^{2}$ is 0.99721. Consequently, the prediction of Young’s modulus using the neural network structure depicted in Table 3 is highly accurate.

**Figure 8:**
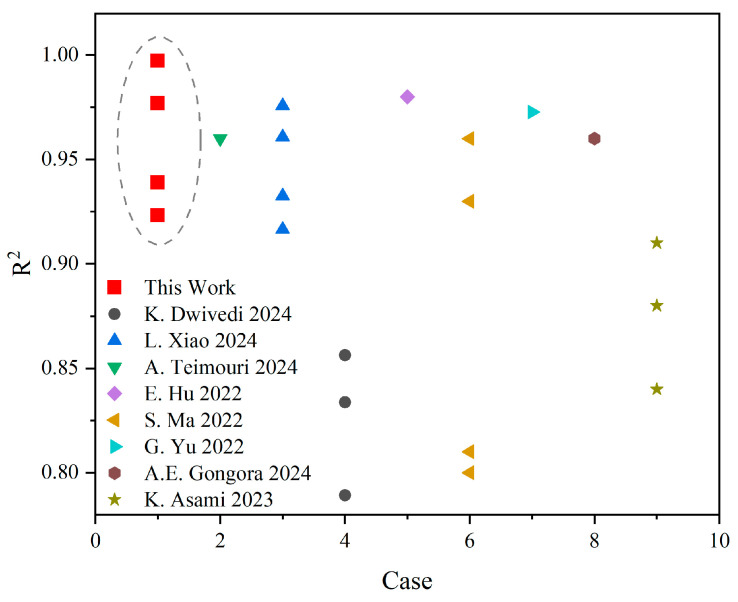
Comparison of the work in this paper with other work [29,34,41,42,43,44,45,46].

In this paper, based on the Gibson–Ashby model, the structure is categorized into two deformation forms, tension and compression dominant and bending dominant, and the above results are obtained by training them separately. Compared with the existing models that are trained directly [29,34,41,42,43,44,45,46], as shown in Figure 8. In the literature on the prediction of mechanical properties of lattice structures, the $R^{2}$ obtained by the classification prediction method used in this paper is better than the rest of the literature, which also shows the necessity of using the Gibson–Ashby model for classification discussion of structures.

### 3.3. ANN Application Deployment

**Figure 9:**
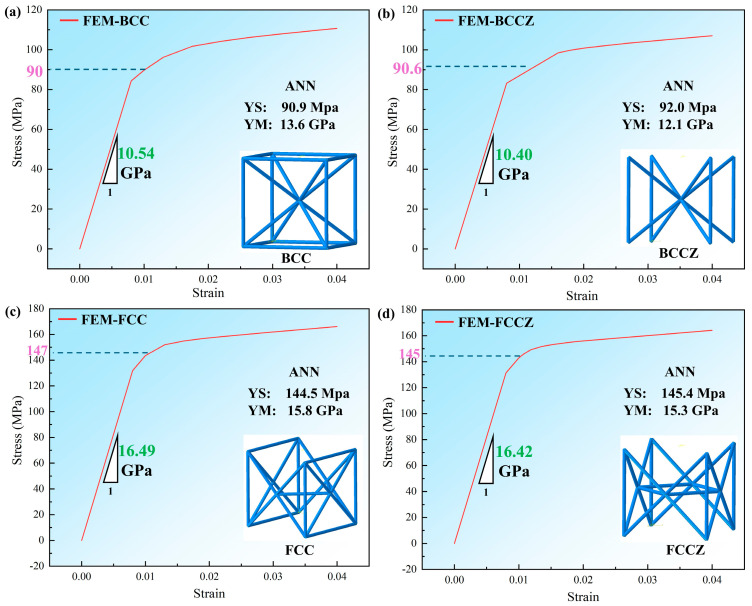
Comparison of Young’s modulus (YM) and yield stress (YS) obtained using ANN with stress−strain curves obtained by simulation (**a**) BCC; (**b**) BCCZ; (**c**) FCC; (**d**) FCCZ.

The establishment of the aforementioned four groups of machine learning models enables the prediction to be carried out for the four more commonly used lattice structure models, namely BCC, BCCZ, FCC, and FCCZ. The Young’s modulus and yield stress are calculated using the ABAQUS 2024 software, and the stress–strain curves of the four structures are obtained, as shown in Figure 9.

In the BCC structure of Figure 9a, Young’s modulus obtained by FEM is 10.54 GPa and the yield strength is 90 MPa, while Young’s modulus obtained by ANN prediction is 13.6 GPa and the yield strength is 90 MPa. Moreover, in Figure 9b, the values of Young’s modulus obtained by FEM and ANN are 10.40 GPa and 12.1 GPa, respectively, and the yield strengths are 90.6 MPa and 92.0 MPa, respectively. A comparison of Young’s modulus and yield strength reveals that the yield strength measured for the BCC structure is more accurate, while the Young’s modulus is predicted to be slightly greater than the Young’s modulus calculated by FEM. Similarly, Figure 9c,d shows that the predicted yield strength values are close to the computed ones, while Young’s modulus values are slightly lower than the computed values in the case of FCC and FCCZ structures. It is worth noting that in this validation process, most considering the model solving time (excluding model creation), the real value obtained by using ABAQUS calculation took 36 s, while the prediction by using ANN took 0.017 s. This result indicates that the prediction efficiency of ANN is much larger than that of finite element calculation.

The preceding results demonstrate the viability of utilizing the model generated through the machine learning method to predict the BCC-FCC lattice structures.

## 4. Conclusions

In order to establish the relationship between Young’s modulus (yield strength) of a typical lattice structure and the topology of the lattice structures, an artificial neural network learning framework for static mechanical properties based on Young’s modulus and yield strength is proposed. This framework is designed to mechanically analyze and learn to predict lattices with different numbers of internal nodes. The following conclusions can be drawn from the research results:

(1) The Gibson–Ashby model was used to fit and analyze the mechanical properties of different lattice structures. The fitting results indicate that the lattice structures mainly have two different deformation modes: a deformation mode dominated by bending and a deformation model dominated by tension and compression. The calculated results for the 4000 structures were found to be mainly divided into two groups. The discrete nature of the results may be attributed to either the lattice structure or the combination of the mixture of the two deformation forms.

(2) It is feasible to utilize the topological form of the lattice structure for the prediction of stress and Young’s modulus. In the validation of both training and test data, the prediction results are within a reasonable margin of error, implying good accuracy. And by calculating $R^{2}$ and comparing it with the rest of the literature, it proves that the computational method provided in this work is superior. Furthermore, the prediction is also effective for typical BCC(Z) and FCC(Z) structures. This also means that the ANN model established in this paper helps to further develop lattice structures, which can promote the wider application of lattice structures.

(3) In this prediction process for the lattice structure, due to the different deformation behaviors of the lattice structure, there are tensile deformation, bending deformation, and mixed deformation modes. This is a great challenge to improve the prediction accuracy. In this paper, it is classified into two different deformation forms according to the G-A model, but it still needs to be improved. In the future research process, a more profound discussion and study is needed for its deformation modes. 

## Figures and Tables

**Table 1:** The dataset of the machine learning model.

No	ML Model	Dataset Type	Input Parameters	Comparison
1	ANN	Topology feature	23	This work
2 [28]	ANN	Geometric feature	3	Poor generalizability to other lattice structures
3 [33]	ANN	Shape features	1000	Too large data inputs for deployment of applications and lightweight operation
4 [32]	NN	Digital visualization	400	Too large data inputs for deployment of applications and lightweight operation
5 [30]	SG-based machine learning	Geometric feature	3	Poor generalizability to other different structures
6 [22]	GNN	Topology feature	55	large data inputs
7 [34]	DNN	Representative volume Element	20	Only for plate–lattice structures

**Table 2:** The dataset of the machine learning model.

Internal Node	Number of Data	Yield Strength (MPa)	Young’s Modulus (MPa)
N1	1000	From FEM	From FEM
N1, N2	1000	From FEM	From FEM
N1, N2, N3	1000	From FEM	From FEM
N1, N2, N3, N4	1000	From FEM	From FEM

**Table 3:** The elasticity and plasticity model used in FEM.

Elastic		Johnson–Cook Plastic		
Young’s Modulus	Poisson’s Ratio	A	B	N
107 GPa	0.3	1567 Mpa	952 Mpa	0.4

**Table 4:** The structures of yield stress prediction group 1.

Group Type	1# Layer	2# Layer	Activation Function	Iterations
$\sigma^{*}=0.52\rho^{*0.94}$	233	253	ReLU	1000
$\sigma^{*}=0.39\rho^{*1.50}$	69	0	Sigmoid	1000

**Table 5:** The structure of Young’s modulus prediction group 1.

Group Type	1# Layer	2# Layer	Activation Function	Iterations
$E^{*}=0.41\rho^{*0.62}$	72	295	ReLU	1000
$E^{*}=0.38\rho^{*0.80}$	300	251	tanh	1000

## Data Availability

The raw data supporting the conclusions of this article will be made available by the authors on request.
